# Ultra Narrow Dual-Band Perfect Absorber Based on a Dielectric−Dielectric−Metal Three-Layer Film Material

**DOI:** 10.3390/mi12121552

**Published:** 2021-12-12

**Authors:** Bin Liu, Pinghui Wu, Hongyang Zhu, Li Lv

**Affiliations:** 1 Rural Revitalization Institute, Linyi University, Linyi 276000, China; liubin@lyu.edu.cn; 2 Center for International Education, Philippine Christian University, Manila 1004, Philippines; 3 Fujian Provincial Key Laboratory for Advanced Micro-Nano Photonics Technology and Devices, Quanzhou Normal University, Quanzhou 362000, China; phwu@zju.edu.cn; 4 School of Physics and Electronic Engineering, Linyi University, Linyi 276000, China

**Keywords:** dielectric, narrowband, metamaterial perfect absorber, high-Q, tunability

## Abstract

This paper proposes a perfect metamaterial absorber based on a dielectric−dielectric−metal structure, which realizes ultra-narrowband dual-band absorption in the near-infrared band. The maximum Q factor is 484. The physical mechanism that causes resonance is hybrid coupling between magnetic polaritons resonance and plasmon resonance. At the same time, the research results show that the intensity of magnetic polaritons resonance is much greater than the intensity of the plasmon resonance. By changing the structural parameters and the incident angle of the light source, it is proven that the absorber is tunable, and the working angle tolerance is 15°. In addition, the sensitivity and figure of merit when used as a refractive index sensor are also analyzed. This design provides a new idea for the design of high-Q optical devices, which can be applied to photon detection, spectral sensing, and other high-Q multispectral fields.

## 1. Introduction

Metamaterials are composite structures made of artificial unit structures, which can realize sensing [1,2], photocatalysis [3,4,5], thermal emitters [6], infrared detection and imaging equipment [7], and other applications [8,9]. Their singular properties are derived from artificially designed microstructures, rather than determined by the composition of the materials. The perfect absorption of waves can be achieved by using metamaterials. The principle is to achieve zero transmission and reflection at the frequency of interest. When the free space impedance is equal to the metamaterial impedance, the reflection is minimal. The traditional metamaterial absorber is generally composed of a metal−dielectric−metal (MDM) structure, and different absorption characteristics are obtained by designing different microstructures on the top metal [10,11,12,13,14,15]. These MDM structures have a general feature for the design of plasmon and metamaterial absorbers, with a thin dielectric interval in between, in order to realize the strong plasmon near-field coupling between the top plasmonic resonator and the bottom metal reflector. Because of the inherent high optical loss of metals, the resonant absorption bandwidths of the absorbers are relatively wide [16,17,18,19,20,21,22,23]. In addition, metals are also easy to corrode and oxidize, their structure is complex, and their preparation costs are relatively high, which limit their applications. Compared with metal materials, dielectric materials have the characteristics of a simple structure, stable performance, and low ohmic loss, which are popular in the research of metamaterials today [24]. In some applications, the difference in absorption bandwidth determines different effects. For example, broadband absorbers can effectively absorb electromagnetic energy in a wide range of wavelengths and can be used in fields such as solar absorbers [25,26,27,28]. The narrow-band absorber has a high time coherence, and this high sensitivity and figure of merit are conducive to applications in refractive index sensors and other fields [29,30,31,32]. Recently, many researchers have conducted a series of studies on narrow-band perfect absorbers based on dielectric materials. For example, Liao et al. recently achieved perfect absorption with an absorption bandwidth of 1.3 nm through a dielectric structure set on a metal substrate [33]. Another example is that by adding a layer of Si medium to the surface of the metal grating to form a complementary structure, Wang et al. proved that adding a layer of Si complementary grating structure could effectively reduce the absorption bandwidth of the resonance peak [34]. However, the narrowest absorption bandwidth they achieved was only 5.4 nm.

This paper designs a dielectric−dielectric−metal (DDM) structure. The bottom metal plate prevents electromagnetic waves from penetrating the structure, so that the transmission is zero. The intermediate dielectric layer was a SiO_2_ plane layer, and the frequency selective surface was a grating structure made of a TiO_2_ dielectric material. The absorber achieves dual-band perfect absorption in the near-infrared range. The resonance peaks are located at 865.295 nm and 967.645 nm, the absorption rates are 97.6% and 99.1%, the absorption bandwidths are 11 nm and 2 nm, and the Q factors are 79 and 484, respectively. We analyzed the physical mechanism of its resonance, and also discussed the tunability of the structure and the tolerance of the working angle. By changing the environment where the absorber is located, its sensing performance could be analyzed. Our design provides a new design idea for high-Q optical devices, which can be applied to a variety of high-Q factor multispectral applications.

## 2. Structure Design and Numerical Model

**Figure 1:**
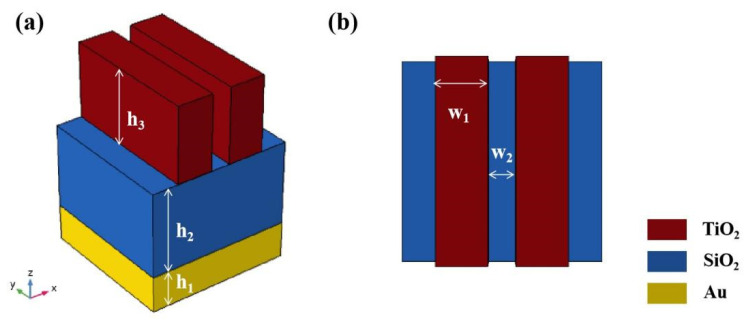
(**a**,**b**) Three-dimensional view and a top view of the designed absorber, respectively.

**Figure 2:**
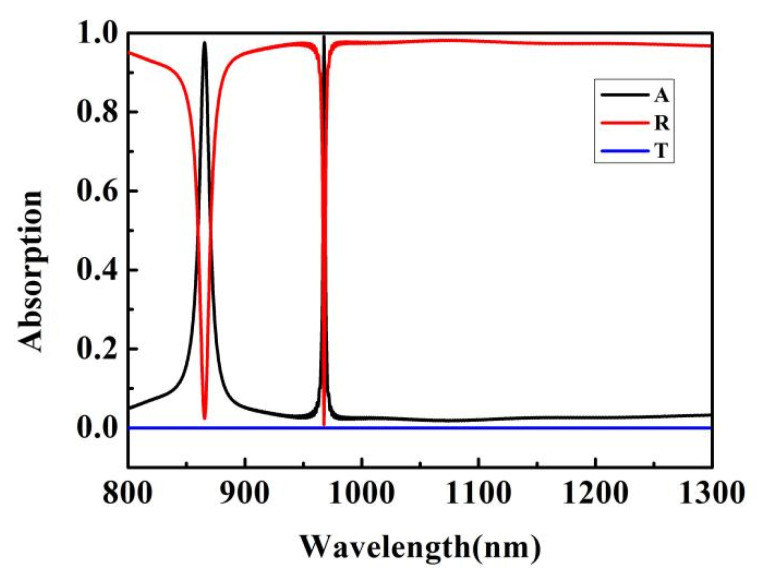
Spectra of R, T, and A under TM polarized light.

Figure 1 shows a schematic diagram of the designed absorber. The substrate adopts a metal plane layer so that the transmission of the structure is 0, as shown in Figure 2. The dielectric property parameters are from the experimental data provided by Johnson and Christy et al. [35]. The intermediate dielectric layer is made of SiO_2_ with a refractive index (RI) of 1.45. The top layer is a dielectric grating structure, using TiO_2_, with a RI of 2.4 [36,37]. As material loss was not considered, the RI of the dielectric material was set to a pure real number. In the figure, the physical meaning of the structural parameters s indicated by letters. We used FDTD Solutions software for the simulation. In the simulation process, the parameters were set as: w_1_ = 150 nm, w_2_ = 80 nm, h_1_ = 150 nm, h_2_ = 350 nm, h_3_ = 313 nm, and px = py = p = 600 nm. By adding time domain monitors, the transmission (T) and reflection (R) data of the structure could be obtained. According to the formula A = 1-R-T, the absorption data could be obtained [38,39,40,41]. In the simulation process, a plane light source was used for the incidence, and the x, y, and z directions were the antisymmetric, symmetric, and PML boundary conditions, respectively.

## 3. Simulations Results and Discussions

Figure 2 shows the transmission, reflection, and absorption spectra of the absorber. It can be seen that this absorber can obtain double-band perfect absorption in the near-infrared band range of 800–1300 nm. The resonance peaks are λ_1_ = 865.295 nm (absorption is 97.6%) and λ_2_ = 967.645 nm (absorption is 99.1%). Their full width at half maximum (FWHM) was 11 nm and 2 nm, and the Q factors were 79 and 484. It achieved ultra-sharp resonance light absorption. The absorber had the characteristics of a high Q factor, simple structure, and easy preparation. It provided a new method for the design of high-Q optical devices, and could be used in various applications, such as photon detection and spectral sensing.

**Figure 3:**
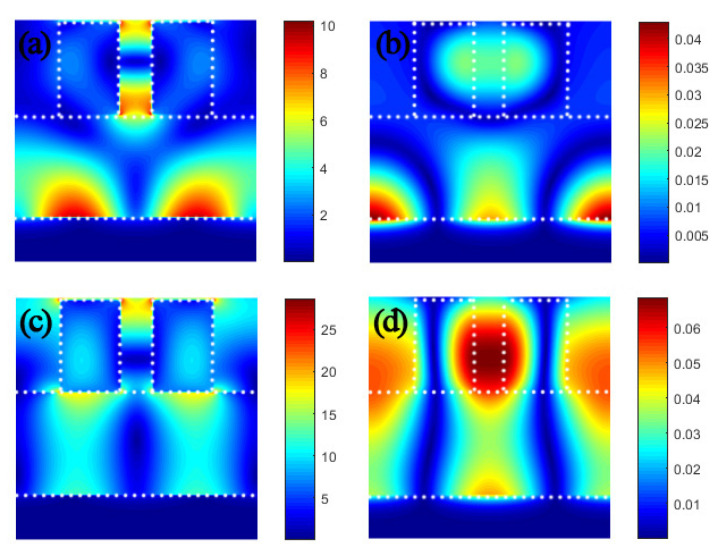
(**a**,**b**) Distribution of electric and magnetic fields at λ_1_ = 865.295 nm. (**c**,**d**) Distribution of electric and magnetic fields at λ_2_ = 967.645 nm. The white dotted lines describe the structure of the absorber.

Next, we calculated the electromagnetic field distribution in the two resonance modes to analyze the resonance mechanism. It can be seen from Figure 3a that the electric field at λ_1_ was mainly distributed between the slits of the dielectric grating and metal surface. This shows that under the excitation of incident light, the dielectric layer excited the plasmon resonance of the metal surface. As the dielectric constants of SiO_2_ and gold are very different, the vertical component of the electric field cannot penetrate deep into the metal, but only exists on the surface of the metal [42,43]. For the electric field distribution in the top layer medium, it is obvious that there are red dots with concentrated energy, which indicates that the dielectric grating structure produces magnetic polaritons (MPs), which is a typical feature of MPs resonance [44,45]. The resonance peak at λ_1_ is mainly derived from the plasmon resonance of the metal. This phenomenon can be confirmed according to the magnetic field distribution in Figure 3b. Figure 3c shows the electric field distribution at λ_2_. It can be clearly seen that in this resonance mode, the electric field is mainly distributed in the dielectric layer and the dielectric grating. The intensity of the plasmon resonance excited at this time is much smaller than the intensity of the medium resonance. The strong resonance peak is mainly derived from the MPs resonance supported by the dielectric material, which can be confirmed by the magnetic field distribution in Figure 3d. Comparing the electromagnetic field distribution and intensity in the two modes, it can be seen that the dielectric resonance intensity is far greater than the plasmon resonance intensity. Therefore, ultra-sharp resonance peaks can be generated in the λ_2_ resonance mode. Therefore, dielectric nanoparticles also support electric resonance in metal particles and support MPs resonance. The hybrid coupling between MPs resonance and the plasmon resonance of metal can produce two sharp absorption peaks.

**Figure 4:**
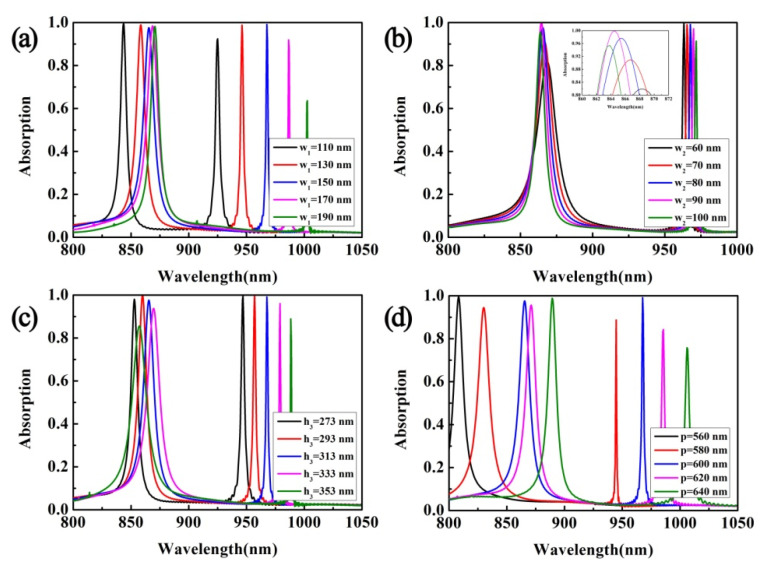
(**a**–**d**) The change of the absorption spectrum when changing the value of w_1_, w_2_, h_3_, and p.

Figure 4a–d shows the change of the absorption spectrum after transforming the geometrical parameters of the metamaterial absorber. Figure 4a shows the change of the absorption spectrum when the width of the grating stripe is changed. As the width increases, both resonance peaks show a red shift. For the resonance peak at λ_1_, the absorption remains basically unchanged, while the absorption at λ_2_ shows a trend of first increasing and then decreasing. Because the resonance peak in this mode is mainly caused by the MPs resonance of the dielectric grating, the width of the grating has a greater influence on the resonance peak at λ_2_. Figure 4b shows the change of the absorption spectrum when the pitch of the grating stripes is changed. As the distance increases, the resonance wavelength at λ_1_ produces a blue shift. The resonance wavelength at λ_2_ produces a red shift, and when the distance exceeds 80 nm, the absorption begins to decrease. Therefore, there is a critical value for the grating fringe pitch. Figure 4c shows the change of the absorption spectrum when the height of the grating stripe is changed. For the resonance peak in the λ_1_ mode, the resonance wavelength first shifts red and then blue as the height increases, and when h_3_ = 353 nm, the absorption rate is the lowest, dropping to about 84%. For the resonant peak in λ_2_ mode, the resonant peak only shows a red shift. When h_3_ changes in the range of 273 nm to 313 nm, perfect absorption can still be maintained. Therefore, the height of the dielectric grating also has a critical value. When it is greater than the critical value, the absorption of the absorption peaks in both modes will be greatly reduced. Figure 4d shows the change of the absorption spectrum when the period of the absorber unit structure is changed. Only when the period is 600 nm, the two absorption peaks can achieve perfect absorption. Interestingly, when the period is 560 nm, only the resonance peak of the λ_1_ mode exists. These results all show that the absorber is tunable, and different absorption functions can be achieved by changing the structural parameters. When the structural parameters are changed, the impedance of the absorber will change, and it cannot match perfectly with the impedance of free space, so it will be manifested by the reduction of absorption or the movement of the absorption peak.

**Figure 5:**
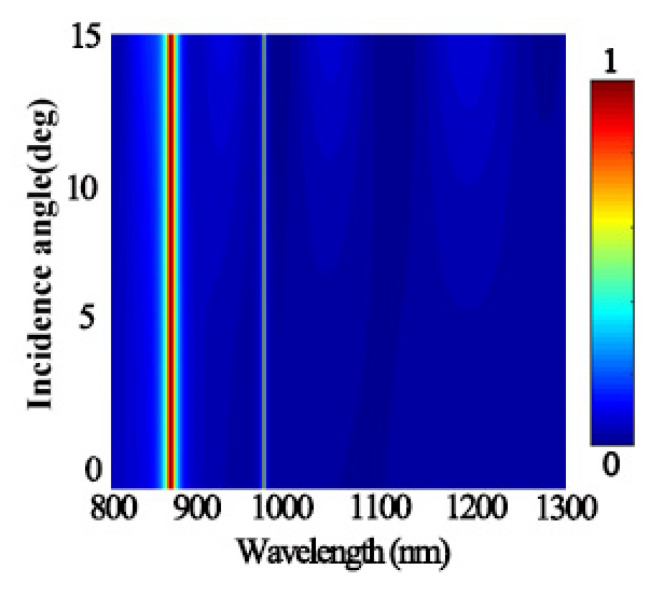
Corresponding resonance wavelengths at different incident angles.

As the designed grating was a one-dimensional structure (extends infinitely along the Y direction), we only studied the working angle tolerance under TM polarized light, as shown in Figure 5. The research results show that the structure could only maintain good absorption characteristics in the range of a 0–15° incident angle, and its absorption peaks position, absorption intensity, and absorption bandwidth remain unchanged. This indicates that within this range, the structure was insensitive to the incident angle.

**Figure 6:**
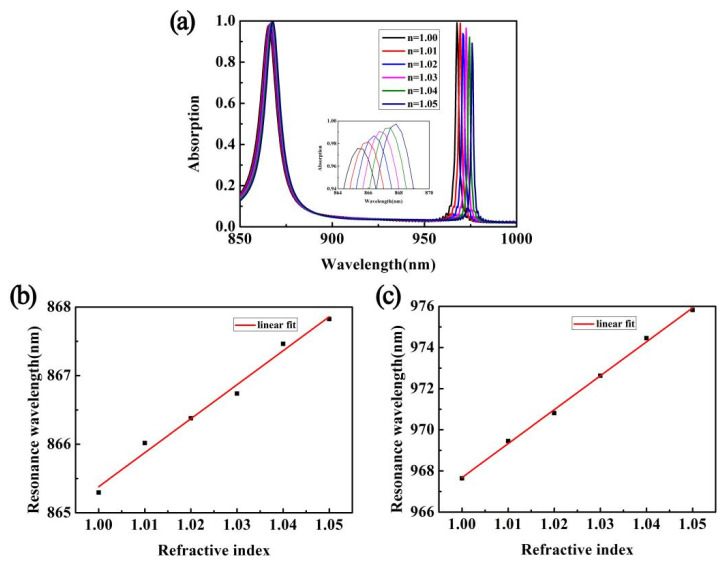
(**a**) The numerical calculation results of the absorption spectrum when the RI of the environment changes. (**b**,**c**) Linear relationships between the resonance wavelength and the RI in the λ_1_ and λ_2_ modes, respectively.

Finally, in order to analyze the sensing performance of the designed dual-band perfect metamaterial absorber, with the other parameters unchanged, we calculated the absorption spectrum under different environmental RIs. The results are shown in Figure 6a. When the RI increases, the resonance peaks at λ_1_ and λ_2_ both show a red shift, the absorption at λ_1_ increases slightly, while the absorption at λ_2_ shows a downward trend. For the resonance peak at λ_1_, the resonance wavelength changes from 865.295 nm to 867.825 nm, and the change is only 2.53 nm. For the resonance peak at λ_2_, the resonance wavelength changes from 967.645 nm to 975.824 nm, and the amount of change is 8.179 nm. The resonance wavelengths of the absorber under different RIs were extracted and numerically fitted. Figure 6b,c shows the results. It can be clearly seen from the figure that for the two resonance modes, there is a linear relationship between their resonance wavelength and RI. Analyzing the sensitivity (S = ∆λ/∆n) and figure of merit (FOM = S/FWHM) of the two modes can get S_1_ = 50 nm/RIU and S_2_ = 165 nm/RIU, and FOM_1_ = 5 1/RIU and FOM_2_ = 83 1/RIU [46,47,48,49,50], respectively. It can be seen that the absorber has a good sensing performance and can be used in refractive index sensors and other fields. Comparing the performance parameters of our proposed absorber with the previous work, as shown in Table 1, it clearly shows the advantage of a high Q factor [51,52,53,54]. Therefore, it is also suitable for various high-Q factor multi-spectral applications, such as photon detection, spectral sensing, and other fields.

## 4. Conclusions

In general, this article achieved dual-band narrowband absorption by designing a dielectric structure on a metal substrate, and obtained a high Q factor of 484. The ultra-sharp light absorption comes from the hybrid coupling between MPs resonance and plasmon resonance. The research results show that the absorber is tunable. When the size and height of the top dielectric grating and the unit structure period are changed, the position and intensity of the resonance peak will change. The maximum working angle tolerance can reach 15°. The sensitivity and FOM of the two resonance modes are S_1_ = 50 nm/RIU and S_2_ = 165 nm/RIU, and FOM_1_ = 5 1/RIU and FOM_2_ = 83 1/RIU, respectively. It has a good sensing performance. It can be applied to high Q factor multi-spectral applications, such as photon detection, sensor filtering, and other fields.

## Figures and Tables

**Table 1:** Comparison of the maximum FOM and Q value of the absorber reported in this paper with the results of the dual-band absorber reported by other works.

Reference	[51]	[52]	[53]	[54]	Proposed
FOM (max) (1/RIU)	16.54	26.67	44.5	12.16	83
Q (max)	19.8	23.33	123.45	71.42	484
